# Analysis of factors contributing to medication errors during self-management of medication in the rehabilitation ward: a case control study

**DOI:** 10.1186/s12913-022-07679-y

**Published:** 2022-03-03

**Authors:** Ryohei Suzuki, Takako Uchiya, Ayumi Nakamura, Naoki Okubo, Takamasa Sakai, Masaaki Takahashi, Mariko Kaneko, Ikuko Aiba, Fumiko Ohtsu

**Affiliations:** 1 Graduate School of Pharmacy, Meijo University, 150 Yagotoyama, Tempaku-ku, Nagoya, Aichi Japan; 2 Department of Pharmacy, National Hospital Organization Higashinagoya National Hospital, 5-101 Umemorizaka, Meito-ku, Nagoya, Aichi Japan; 3 Department of Nursing, National Hospital Organization Higashinagoya National Hospital, 5-101 Umemorizaka, Meito-ku, Nagoya, Aichi Japan; 4 Drug Informatics, Faculty of Pharmacy, Meijo University, 150 Yagotoyama, Tempaku-ku, Nagoya, Aichi Japan; 5 Department of Orthopedics, National Hospital Organization Higashinagoya National Hospital, 5-101 Umemorizaka, Meito-ku, Nagoya, Aichi Japan; 6 Department of Neurology, National Hospital Organization Higashinagoya National Hospital, 5-101 Umemorizaka, Meito-ku, Nagoya, Aichi Japan

**Keywords:** Medication error, Self-administration of medication, Rehabilitation ward, Medication factor

## Abstract

**Background:**

In the rehabilitation ward, many elderly patients require continuous use of medication after a stroke or bone fracture, even after discharge. They are encouraged to self-manage their medications from the time of admission. Medication errors, such as a missed dose or incorrect administered medication can worsen conditions, resulting in recurrent strokes, fractures, or adverse effects. The study was aimed to identify risk factors, such as medication and prescription, contributing to errors in self-management of medication.

**Methods:**

This study was conducted on patients who self-managed their medication in the rehabilitation ward of Higashinagoya National Hospital from April 2018 to March 2020. The patient background including age and sex were investigated. The medication factors examined include the number of medications and administrations per day, dosing frequency on indicated days, prescription and start date are the same, medications from multiple prescriptions, and one package or one tablet at each dosage. The group of medication error cases were defined as the medication error group and that of control cases as the no-medication error group. A logistic regression analysis was performed for factors related to medication errors.

**Results:**

A total of 348 patients were included in the study, of which 154 patients made medication errors, with 374 total medication error cases. The median number of medications in the medication error group was six, and that in the no-medication error group was five. Statistically significant factors correlated with errors made during self-management of medication were the number of medications, number of administrations per day, dosing frequency on indicated days, and medication from multiple prescriptions.

**Conclusions:**

When a patient is self-managing their medications, errors are likely to occur due to a high number of medicines they are taking and the complexity of the dosage regimen. Therefore, to prevent medication errors, reviewing the prescribed medications and devise ways to simplify the dosage regimens is crucial.

## Background

Patients whose symptoms stabilize after acute stroke treatment or fracture surgery are admitted to the rehabilitation ward. They are mainly treated through rehabilitation to improve and/or maintain their activities of daily living (ADL) so they can return to society. In Japan, rehabilitation wards allow long-term hospitalization for up to 180 days, depending on the target disease and severity of conditions. Many of the patients admitted to rehabilitation wards are elderly and/or have underlying conditions. They need continuous medication to prevent recurrent strokes or fractures and to maintain and/or prevent exacerbations of their comorbid conditions. Therefore, appropriate management of medication is required.

There are two types of medication management in hospitals: management by patients and management by medical professionals, mainly nurses. In cases where the patient is incapable to self-manage their medication due to deterioration of their condition or decline in their cognitive functioning, or when they are taking medication that requires careful management, such as narcotics, nurses, and other medical professionals often perform this task. In contrast, self-management of medication is a method that is being implemented as the number of patients with chronic diseases and the need for continuous treatment is increasing with the aging of society. In addition, self-management of medication is conducted because patients themselves wish to face their own diseases and manage their own medications in their pharmacotherapy. Patients in this group practice medication management as preparation for life after discharge. It is necessary to accurately manage medication, but medication errors, such as missed doses or taking the wrong medication have been reported.

In outpatient self-management, the relationship between adherence, medication errors, and the Medication Regimen Complexity Index (MRCI), which is a measure of the complexity of prescriptions consisting of the number of medications and the number of administrations per day, which contributed to the medication error, has been reported [1–5]. It has also been reported that unused medication that remains after patients forget to take medication by self-administration leads to increased medical costs and has become a health economics problem [6, 7]. However, it was reported that self-management of medication led to a reduction in nursing time used check and administration [8], and cost savings due to time savings [9]. And self-management in the hospital setting has been shown to be beneficial in improving patient satisfaction, adherence to medication, and self-care skills [10]. Self-administration has been reported to have a lower rate of medication errors than that of administration by nurses [11]. Although, medication errors occurred in self-management.

Studies on the analysis of risk factors, such as prescription background and medication, are lacking. Understanding the risk factors correlated with medication errors is important for improving the quality of medical care. This may prevent errors from occurring and recurring through self-management. This study was aimed to identify risk factors, such as medication and prescription, contributing to error in self-management of medication.

## Methods

### Methods of medication self-management

In the rehabilitation ward of Higashinagoya National Hospital, physicians, pharmacists, and nurses check the type and number of medications patients are administered, their Functional Independence Measure (FIM) [12], and a Mini-Mental State Examination (MMSE) [13] from the perspective of medical safety. A self-management training was conducted when patients were capable of taking medication. When starting self-management, pharmacists and nurses provided information such as the name of the medication, its purpose, the number of administrations, the timing, and adverse effects. We also trained patients in self-management by ensuring that they can open their medications on their own and understand when to take them appropriately. We gave them press-through pack and/or one packaged medication, in which medications are placed in bags or other container with dosing schedules. In this study, self-management of medication was defined as self-management of more than 1 day’s worth of medication. Based on the American Society of Health-System Pharmacists classification system [14], medication errors during self-management were defined as omitted medication, taking the incorrect medication, taking it at an incorrect time, and taking an incorrect dose.

### Methods of recording medication error cases

The way to check for medication errors was to let the patient who was self-managing leave the package of the medication after taking it. Nurses subsequently checked this package at each dosage time to ensure that the correct medication and dose was taken at the correct time, as prescribed during the self-management training and confirmed a medication error. In addition, when a medication error occurred, the nurse recorded the details of the errors.

### Survey targets and methods for extracting medication error cases

In this study we included self-management medication error cases that occurred in a rehabilitation ward between April 1, 2018, and March 31, 2020. We extracted these cases by reviewing all nursing records during the study period.

### Research items

This study retrospectively surveyed patient background and medication factors based on electronic medical records and dispensing records, such as prescriptions.

#### Patient background

This study surveyed the following aspects of patient background from the electronic medical records: age, sex, primary medical department, length of self-management of medication, and length of hospital stay. This study was also surveyed the MMSE score, functional independence measure-motor (FIM-M) score, which is the sum of the scores of 13 items related to exercise, and functional independence measure cognition (FIM-C) score, which is the sum of the scores of five items related to cognition.

#### Medication factors

We surveyed the following medication factors from electronic medical records and dispensing records: number of medications [4, 15], number of administrations per day [4, 15], dosing frequency on indicated days [16], prescription and start date are the same, orders prescribed by more than one physician and/or by the same physician at different times (medication from multiple prescriptions), continuous use of medications received prior to admission, and not one package [17] or tablet at each dosage.

### Research design and statistical methods

We conducted a case-control study to analyze factors correlated with medication errors. A patient who made a medication error was considered the case patient. In addition, as one patient could make multiple medication errors, each medication error occurring during medication self-management within the period of study was considered as one medication error case. Then a patient who didn’t make a medication error during their hospitalization was considered the control patient. The corresponding cases were defined as case of a control patient, the control cases were randomly selected from control patients who were admitted to the same rehabilitation ward at the time the medication error case was confirmed. If a patient made multiple medication errors, a control case was included for each medication error case. The group with medication error was defined as the medication error group, and the group with control cases was defined as the no-medication error group.

We compared patient backgrounds and medication factors between patients who made a medication error (the medication error group) and those who did not (no-medication error group) using univariate logistic regression analysis. In addition, a multivariate logistic regression analysis was performed by selecting medication factors (*p* < 0.2 in univariate logistic regression analysis). We confirmed the variance inflation factors (VIF), which are indicators of multicollinearity.

We also calculated medication factors that were statistically significantly correlated in multivariate logistic analysis for the medication error and no-medication error groups. This study compared the number of medication factors in the medication error and no-medication error groups using the Mann–Whitney U test. The number of medications was a risk factor if the number of medications was five or more from the aspect of polypharmacy [18], and if the number of administrations per day was three or more from the aspect of adherence [19].

The significance level was set at 5%. IBM SPSS Statistics Version 27 (IBM Corporation) was used to analyze the data.

### Ethical considerations

This study was approved by the Ethical Review Committee of the Higashinagoya National Hospital.

## Results

### Patient backgrounds

A total of 348 patients self-administered their medications during the study period. The medication error group comprised of 154 patients who had medication errors and 374 cases of medication errors. A total of 194 patients did not make medication errors and 374 cases in the no-medication error group. The case group constituted 84 patients with multiple medication errors and 304 medication error cases.

The backgrounds of the 154 and 194 patients who made and did not make medication errors are shown in Table 1. The median age of the patients who made a medication error was 76 years, with 79 men and 75 women in this group. Furthermore, the median age of patients who did not make medication error was 75 years, consisting of 84 men and 110 women in this group. When comparing the patient backgrounds of each group, age, sex, FIM-M, FIM-C, or MMSE scores were not statistically significantly different. However, compared to the patients who did not make medication errors, those who made medication errors showed a statistically significant increase in the duration of the hospital stay and medication via self-management.

**Table 1:** Patient background

	Patients who made a medication error (*n* = 154)		Patients who did not make a medication error (*n* = 194)		*P*
Age, median (IQR), years	76	(65–82)	75	(65–82)	0.65 ^a)^
sex, n (%)					
men	79	(51)	84	(43)	0.14^b)^
women	75	(49)	110	(57)	
Length of hospital stay, median (IQR), day	48	(34–71)	42	(24–57)	0.01 ^a)^
Length of self-administration of medication, median (IQR), day	34	(24–57)	27	(15–44)	< 0.01 ^a)^
Primary medical department, n (%)					
Neurology	65	(42)	79	(41)	0.78 ^b)^
Orthopedic Surgery	60	(39)	81	(42)	0.60 ^b)^
Neurosurgery	29	(19)	34	(18)	0.75 ^b)^
MMSE, median, (IQR), score	28	(25–29)	27	(25–29)	0.38 ^a)^
Unknown, n (%)	18	(12)	44	(23)	
FIM, median, (IQR), score					
FIM-M					
admission	61	(52–70)	64	(52–72)	0.37 ^a)^
discharge	83	(76–87)	83	(77–87)	0.37 ^a)^
FIM-C					
admission	31	(28–34)	31	(28–34)	0.40^a)^
discharge	34	(31–35)	34	(31–35)	0.61 ^a)^

*IQR* Interquartile range, *MMSE* Mini-Mental State Examination, *FIM-M* Functional Independence Measure-Motor, *FIM-C* Functional Independence Measure-Cognition, a) Mann–Whitney U test, b) χ^2^ test

### Medication factors

The medication factors for each group are shown in Table 2. The median number of medications in the medication error group was six, and the median number of administrations per day was three. In the no-medication error group, the median number of medications was five, and the median number of administrations per day was two. The medication factors that exhibited *p* < 0.2 in the univariate analysis were the number of medications, number of administrations per day, dosing frequency on indicated days, medication from multiple prescriptions, and not one package or one tablet at each dosage. The results of the multivariate logistic regression analysis of medication factors are shown in Table 3. The medication factors that showed a statistically significant correlation in the multivariate logistic regression analysis were the number of medications, number of administrations per day, dosing frequency on indicated days, and medication from multiple prescriptions. The VIF was less than two for the medication factors, and multicollinearity was not observed.

**Table 2:** Medication factors

	Medication error group (*n* = 374)		No-medication error group (*n* = 374)		*P*
Number of medications, median (IQR)	6	(4–8)	5	(3–7)	< 0.01 ^a)^
Number of administrations per day, median (IQR)	3	(2–4)	2	(1–3)	< 0.01 ^a)^
Dosing frequency on indicated days, n (%)	22	(6)	8	(2)	< 0.01 ^b)^
Prescription and start date are same, n (%)	10	(3)	14	(4)	0.41 ^b)^
Medication from multiple prescription, n (%)	194	(52)	124	(33)	< 0.01 ^b)^
Continuous use of medication received prior to admission, n (%)	62	(17)	63	(17)	0.92 ^b)^
Not one package or one table at each dosage, n (%)	287	(77)	250	(67)	< 0.01 ^b)^

*IQR* Interquartile range, a) Mann–Whitney U test, b) χ^2^ test

**Table 3:** Multivariate logistic regression analysis of medication factors

	Medication error group (*n* = 374)		No-medication error group (*n* = 374)		Odds Ratio	95%CI	*P*
Number of medications, median (IQR)	6	(4–8)	5	(3–7)	1.1	1.0–1.2	0.01
Number of administrations per day, median (IQR)	3	(2–4)	2	(1–3)	1.1	1.0–1.3	0.04
Dosing frequency on indicated days, n (%)	22	(6)	8	(2)	2.6	1.1–6.2	0.03
Medication from multiple prescriptions, n (%)	194	(52)	124	(33)	1.8	1.3–2.5	< 0.01
Not one package or one table at each dosage, n (%)	287	(77)	250	(67)	0.7	0.5–1.1	0.13

*IQR* Interquartile range

### Number of medication factors that were statistically significantly correlated with medication errors

The medication factors that showed statistically significantly correlation in the multivariate logistic regression analysis were identified as number of medications, number of administrations per day, dosing frequency on indicated days, and medication from multiple prescriptions. The median number of medication factors in the medication-error group was two, which was statistically significantly higher than that in the no-medication error group, which was one (Table 4).

**Table 4:** Number of medication factors that were statistically significantly correlated with medication errors

	Medication error group	No-medication error group	*P*
Number of medication factors, median (IQR)	2 (1–3)	1 (0–2)	< 0.01 ^a)^

*IQR* Interquartile range, a) Mann–Whitney U test

## Discussion

This study aimed to identify risk factors associated with medication error during self-management. It was found that the risk factor, such as the number of medications, number of administrations per day, dosing frequency on indicated days, and medication from multiple prescriptions were statistically significantly correlated with medication error. In this study, there was no difference in patient background with respect to age, sex, cognitive function, or motor function, except for the length of hospital stay and the length of medication self-management. This suggests that the influence of medication factors is more important than patient background when patients make errors while self-managing their medication.

The median number of medications in the medication-error group was six. There was a statistically significantly correlation between the number of medications taken and medication errors, as previously reported [4, 5]. The high number of medications might be a factor influencing medication errors due to their complicated management. The median number of administrations per day in the medication error group was three which was statistically significantly higher than that in the no-medication error group. It has been reported that adherence to medication when administered over three times or more per day is lower than that of medications taken once a day [19]. The results of this study were similar to those reported in a previous study [19]. This may be because as the number of administrations per day increases, there are more opportunities for medication errors, such as missed doses or taking the wrong medication. Elderly patients often suffer from multiple diseases and are likely to require multiple medications [20]. This leads to a high number of medications and the number of administrations per day. Therefore, it is necessary to review medications before discharge, considering the patient’s life history. In addition, if patients are taking medications that can be discontinued as their symptoms and laboratory values improve, the medications should be reviewed.

Furthermore, the dosing frequency on indicated days was statistically significantly correlated with medication errors. Previous reports have shown that medication adherence was higher with once-weekly medications than with daily medications. However, these studies were based on self-reporting and medication possession ratios [21, 22]. Therefore, it was not possible to confirm whether the medication was administered on a specified day. In this study, medication confirmation was performed by nurses at each dosage, and it could be detected if a dose was missed or wrong medication was taken, dosing frequency on indicated days of medication leads to medication error. In addition, the patients in this study took multiple medications daily, which included weekly and alternate doses. This may lead to a decrease in attention and awareness of how to take these medications, which could be a factor responsible for missed doses.

The presence of medications from multiple prescriptions was statistically significantly correlated with medication errors. As the condition of the patient changes during hospitalization, additional medications are added by the attending physician and other physicians, resulting in multiple prescription configurations that must be taken at one time. As a result, the complexity of medication management is considered a factor in medication errors.

The number of medication factors in the medication error group was statistically significantly higher than that in the no-medication error group. The MRCI is an indicator of prescribing complexity, but the MRCI is complicated to score and does not include medication factors, such as medication from multiple prescriptions. Therefore, the number of medication factors that are statistically significantly correlated with medication errors can be easily used to predict the risk of medication errors.

One limitation of the study was that it relied on nurses’ records, which might lead to underreporting of medication errors due to omissions and mistakes. This study was not able to assess patients’ knowledge and understanding of their medications. However, there was no difference in patient background at the time of admission and discharge, such as FIM-M and FIM-C. Although data deficits were present in the MMSE, it has been shown that cognitive measures such as the MMSE are correlated with FIM-C [23]. As there were no differences in the MMSE scores, the effect of differences in cognitive ability was considered to be small. Therefore, it is thought that medication errors when self-managing one’s medication are influenced by risk factors, such as the number of medications, the number of administrations per day, and medication from multiple prescriptions. Therefore, physicians, pharmacists, and nurses cooperatively need to reduce these factors after confirming the patient’s condition and social background. In addition, this survey only included patients in the rehabilitation ward, whose background may differ from that of patients with other diseases. Therefore, in the future, it will be necessary to conduct a survey regardless of the disease. In addition, the length of hospital stay, and medication self-management were longer in the medication error group. This may have increased the opportunity for medication errors, leading to their occurrence. Another limitation of the study was that the length of self-management was not considered a factor. This study was, for the first time, able to identify the risk factors of medication errors. We believe that these factors could be used to improve the medical treatment of the patient and, thereby, medication safety.

## Conclusions

This study identified risk factors contributing to medication errors during medication self-management; these were the number of medications, number of administrations per day, dosing frequency on indicated days, and medication from multiple prescriptions. To prevent the occurrence of future medication errors, it is necessary to review these factors.

## Data Availability

The datasets used and/or analyzed during the current study are available from the corresponding author upon reasonable request.

## Abbreviations

ADL: Activities of daily living
FIM: Functional Independence Measure
FIM-M: Functional Independence Measure-Motor
FIM-C: Functional Independence Measure Cognition
MMSE: Mini-Mental State Examination
MRCI: Medication Regimen Complexity Index
VIF: Variance Inflation Factors
